# Qualitative study exploring perceptions, attitudes and practices of adolescent university students in Lagos, Nigeria, towards a healthy lifestyle

**DOI:** 10.4102/phcfm.v14i1.3577

**Published:** 2022-10-18

**Authors:** Nnamdi C. Menakaya, Ifeoma N. Menakaya

**Affiliations:** 1 Medicine and Occupational Health Clinic, 11PLC Clinic, Lagos, Nigeria; 2 Department of Restorative Dentistry, Faculty of Dentistry, Lagos State University College of Medicine, Lagos, Nigeria

**Keywords:** adolescent, universities, Nigeria, students, healthy lifestyle

## Abstract

**Background:**

Lifestyle behaviours such as physical activity, dietary habits and sedentary behaviour are developed and established during adolescent years.

**Aim:**

This study aimed to explore the perceptions, attitudes and practices of adolescent university students in Lagos, Nigeria, towards healthy lifestyle.

**Setting:**

Three university institutions in Lagos, Nigeria.

**Methods:**

Qualitative research design was employed. In-depth interviews (IDIs) using an audio-recorder were conducted with 18 adolescent university students recruited through purposive sampling from three university institutions in Lagos, Nigeria. Recorded interviews were transcribed verbatim and thematic content analysis was used to analyse transcribed data.

**Results:**

Study participants perceived a healthy lifestyle as balanced diet, regular physical activity and disease prevention. They described their attitudes and practices towards a healthy lifestyle in terms of personal conviction and disposition, regular physical activity and consumption of fruits and vegetables. Meal skipping and junk food consumption were notable unhealthy dietary practices. Home environment, school campus physical environment, peers, secondary school background and concerns about fast food quality were positive influences towards healthy lifestyle, while academic schedules, personal disposition school campus eateries and sociocultural background were negative influences. Computer and Internet activity was the main sedentary activity.

**Conclusion:**

Study participants’ perception of healthy lifestyles in terms of balanced diet and physical activity aligned with the evidence-based guidelines for healthy lifestyle concepts. Regulatory policies and built environment that promote healthy dietary practices among adolescent university students are recommended.

**Contribution:**

Future research is suggested to examine the benefit of study participants’ engagement in computer or Internet sedentary activities for academic activity and knowledge seeking towards healthy lifestyles.

## Introduction

Healthy lifestyle has been defined as a way of living that reduces the risk of being seriously ill or dying early.^1^ Patterns of dietary intake, physical activity and inactivity, tobacco smoking and alcohol consumption are healthy lifestyle indices that have been universally emphasised and accepted as central to evidence-based guidelines for children, adolescents and adults from diverse populations.^2^

Adolescents are young people in the age bracket of 10 – 19 years.^3^ Lifestyle behaviours such as physical activity, dietary habits and sedentary behaviours are developed and established during adolescent years.^4^ Vulnerability to risky behaviours is characteristic of adolescence and could have significant consequences for future health and well-being.^5^ Prevalence of obesity and overweight has been observed to be increasing among adolescents and has been attributed to environmental factors and behaviours related to diet and physical activity.^6^ Overweight and obesity in adolescence may be associated with risks of developing hypertension, diabetes mellitus and cardiovascular diseases in adult life.^6,7^ A study in Hong Kong also showed a longitudinal association between central obesity and dental caries in 15 – 18-year-old adolescents.^8^

Related studies from Nigeria have mainly been quantitative, involving secondary school student populations.^9,10,11^ These studies found low levels of physical activity,^9^ high levels of sedentary behaviour^9^ and unhealthy dietary practices^10^ among private secondary school adolescents. Qualitative studies from other parts of Africa have also reported on secondary school student populations.^12,13,14^

This study explored the perceptions, attitudes and practices of adolescent university students towards healthy lifestyles in order to elucidate the factors that influence their healthy lifestyle choices. Gaining an understanding of the perspectives of adolescent university students will add to the existing body of knowledge on the subject. Moreover, findings from this study could help to suggest appropriate health promotion strategies for preventing unhealthy lifestyle-related risk behaviours among adolescent undergraduates.

## Materials and methods

### Setting

The study setting involved two public universities and one private, namely Lagos State University (LASU), the University of Lagos (UNILAG) and Caleb University. Lagos State University is owned by the Lagos State government and situated in Ojo, an urban community in the south-western part of Lagos. It has a three-campus setting, and study participants were recruited from two of the three campuses, namely the main campus at Ojo and the College of Medicine campus in Ikeja, the Lagos State capital. The private university institution, Caleb University, has a single campus setting located in Imota, a rural suburb situated in the north-eastern part of Lagos. The UNILAG, which provided two study participants recruited for the pilot study, is one of the pioneer federal universities in Nigeria. It has a multicampus setting with the main campus at Akoka and the College of Medicine campus at Idi-Araba. Both campuses are situated in the mainland part of Lagos. The two study participants from UNILAG were from the College of Medicine Campus in Idi-Araba.

Although Lagos is basically a Yoruba-speaking environment (Yoruba is one of Nigeria’s three major ethnic groups), it has a cosmopolitan and multicultural setting that hosts millions of Nigerians from diverse ethnic backgrounds. This multicultural nature of Lagos is reflected in the multi-ethnic background of students attending university institutions in Lagos, including the participants in this study.

### Study design

A qualitative study design was employed in this study to gain understanding into how and why study participants perceive and live out their experiences in relation to their perceptions, attitudes and practices towards a healthy lifestyle.

The study employed a total of 18 in-depth interviews (IDIs), which were conducted by the lead author, each lasting for about 48 min. The rationale for using 18 IDIs was based on findings of previous studies suggesting that data saturation is reached at about the 12 interview.^15^ The interviews were conducted with 18 adolescents from three university institutions in Lagos, Nigeria, selected by purposive sampling between 23 March 2014 and 07 June 2014.

In this study, balanced diet, personal hygiene, regular fruit and vegetable consumption, infrequent fruit and vegetable consumption, physical exercise, physical fitness, breakfast skipping, infrequent patronage of fast food joints, junk food consumption, computer and Internet activity, staircase climbing, daily domestic chores, academic schedule, home environment, school environment, personal disposition, peers, frequent computer or Internet activity and infrequent television (TV) viewing were recurring codes consistent with the concept of the study objectives in 12 IDIs.

### Inclusion criteria

Study participants who were fluent in English were included. Equivalent proportions of male and female adolescents aged 18–19 years attending each of the three university institutions, namely Caleb University (eight study participants), LASU (eight study participants) and UNILAG (two pilot study participants) were recruited for the study (Table 1). Caleb University and LASU were chosen because while LASU is the only state government–owned public university in Lagos, Caleb University is the only privately owned university in Lagos. Two adolescents (male and female, both aged 18 years) from the federal government–owned UNILAG who satisfied the study recruitment criteria were included as part of the main study, and they were also selected by purposive sampling for a pilot study to pretest the interview guide. A focus group discussion study exploring the determinants of eating behaviour in Belgian university students conducted a pilot study on 10 participants to pretest the study interview guide questions, and with no resultant modifications made to the interview guide, the pilot study data were included as part of the study analysis.^16^ The pilot study data were similarly included in the analysis of the main study because the same interview guide was used for both the pilot study and the main study with no modifications.

**TABLE 1:** Study participants’ demographics.

Label or number code		Course of study	Study level	Institution	Father’s and mother’s occupations	Interview length (minutes)
Age	Gender					

Ethnicity	
**Pilot study interviews**						
CVM1995 or T1		Physiology	Year 1	UNILAG	Patent medicine retailer, caterer	51
18	Female					
Ibo						
SOO1995 or T2		Medicine	Year 3	UNILAG	Lawyer, lawyer	46
18	Male					
Yoruba						
**Main study interviews**						
HIA1995 or P1		Medicine	Year 3	LASU	Teacher, nurse	41
19	Female					
Yoruba						
MEA1996 or P2		Medicine	Year 3	LASU	Doctor, health educator	46
18	Female					
Ora						
TJO1995 or P3		Law	Year 1	LASU	Clergy, teacher	50
19	Male					
Yoruba						
OEA1995 or P5		Law	Year 1	LASU	Clergy, teacher	48
18	Male					
Yoruba						
OEA1995 or P5		Microbiology	Year 2	LASU	Business, teacher	47
19	Female					
Yoruba						
OEA0994 or P6†		Computer Science	Year 3	Caleb	Accountant	51
19	Male					
Yoruba						
OJE1996 or P7		Computer Science	Year 3	Caleb	Publisher, business	46
18	Male					
Ibo						
VAS0994 or P8†		English	Year 3	LASU	Business	48
19	Female					
Egun						
AAA1995 or P9		History, Int’l Relat.	Year 3	LASU	Accountant, teacher	47
18	Male					
Yoruba						
ELE1294 or P10		Architecture	Year 3	Caleb	Surveyor, clergy	47
19	Female					
Ugep						
DEF1996 or P11		Mass Comm.	Year 2	Caleb	School principal, business	54
18	Female					
Bini						
SOA1995 or P12		Computer Science	Year 3	Caleb	Engineer, banker	48
18	Male					
Yoruba						
KTO0894 or P13		Architecture	Year 3	Caleb	Surveyor, business	46
19	Female					
Yoruba						
POO1996 or P14		Mass Comm.	Year 2	Caleb	University lecturer, business	48
18	Female					
Edo						
OOO1194 or P15		Architecture	Year 3	Caleb	Lawyer, civil servant	45
19	Male					
Yoruba						
NGJ1995 or P16		Medicine	Year 3	LASU	Retired civil servant, civil servant	48
19	Male					
Yoruba						

Label, alphanumeric label; Caleb, Caleb University; Int’l Relat., international relations; LASU, Lagos State University; Mass Comm., Mass Communications; UNILAG, University of Lagos.

† , P6 and P8’s mothers are deceased.

P1, P2 and P3 have Islam as their religion. The remaining participants are of the Christian faith.

Older adolescents in the age bracket of 18 – 19 years were recruited because according to Nigerian law, an adolescent below the age of 18 years is considered a minor (High Court of Lagos State Civil Procedure Rules),^17^ while 19 years is the upper limit of age bracket for adolescents.^3^ The study participants were therefore competent to sign informed consent prior to the study.

### Sampling technique

Purposive sampling was used to recruit study participants who met the inclusion criteria.^18^ Potential study participants were referred by their peers, who the lead author came in contact with at his local church and his place of work, where some of the peers of the study participants were on a student internship programme. The referred potential study participants were first contacted by the lead author through phone calls. The lead author subsequently made physical contact with them, and referred potential study participants who met the inclusion criteria were given a copy of the participant information sheet (PIS) containing a detailed explanation of the purpose of the study. After perusing the PIS, each potential study participant who volunteered to participate in the study was subsequently contacted at his or her preferred location for the study interview, which was carried out face-to-face by the lead author. Prior to the study interview sessions, each volunteering study participant was given an informed written consent form to sign by the lead author after confirming individually from each of them they clearly understood the contents of the PIS and the voluntary nature of study participation. Participants in this study received no incentives for their participation.

### Data collection

Patterns of dietary intake, physical activity and inactivity, tobacco smoking and alcohol consumption are healthy lifestyle indices universally emphasised and accepted as central to evidence-based guidelines for children, adolescents and adults from diverse populations.^2^ This study focused on three indices, namely dietary, physical activity and sedentary behaviour (physical inactivity). Tobacco smoking and alcohol consumption habits of adolescents in Nigeria vary and are not common indices for evaluating healthy lifestyle among the adolescent population.^19,20,21^ For the purpose of gathering data related to the study objectives, an audio-recorder was used to record the IDIs with the study participants, using a pretested interview guide. The average duration of the interview sessions with individual study participants was 48 min (Table 1). Each interview session was carried out with an assurance of strict confidentiality in handling the information provided. In order to ensure privacy, the interviews were held at each study participant’s location of choice. Alphanumeric identification labels instead of names were employed to further assure confidentiality and anonymity of participants during the transcription of the interview data. Recorded interview data, including nonverbal signals, were transcribed verbatim for purposes of data analysis. Also, the recorded data were stored in the lead author’s secured personal laptop computer, which was accessible only to the two authors.

### Interview guide

The interview guide was designed as open-ended questions. It was developed from a review of existing literature of studies that shared the same theoretical frameworks that informed this study.^4,16,22,23^ Semi-structured IDIs are the most frequently used qualitative data source in health services research.^24^ This method usually consists of a dialogue between researcher and participant, guided by a flexible interview protocol and supplemented by follow-up questions.^24^ It allows the researcher to collect open-ended data and explore the thoughts, feelings and beliefs of study participants on a particular topic.^24^ In this study, the interview guide consisted of open-ended flexible questions related to the study objectives, which allowed for follow-up questions on the basis of study participants’ responses during the interview sessions. Furthermore, open-ended questions offered the advantage of eliciting responses that were informative and explanatory for study participants, meaningful to the participant and unanticipated by the researcher.^18^

The interview guide was approved by the University of Liverpool Research Ethics Committee. A pilot study involving IDIs of two adolescents (male and female, both aged 18 years) referred by their peers who were members of the authors’ local church and neighbourhood, respectively, and selected by purposive sampling was conducted to pretest the interview guide. The pilot study participants were students of the federal government–owned UNILAG who satisfied the study recruitment criteria (Table 1).

According to the social construction theory framework,^22^ the goal of research is to see how the study participants make sense of their experiences. This theoretical framework conceptualises that there is not a single view or truth and that a range of views can be valid in different ways. People construct evidence through their own experiences, and the researcher attempts to elucidate how individuals make sense of their experiences. The words and gestures during interactions are evaluated for how they symbolise bigger issues. Research within a social construction framework also takes into account the expectations, values, backgrounds and roles of the main groups concerned and how meanings of people’s experiences are expressed, perceived and reconstructed. This study assumed the social interactionism type of social theory with the key concepts of social groups, social interactions, interpretation and meaning.^23^

### Data credibility, transferability, confirmability and dependability

In this study, a purposive sampling approach was employed that sought to identify specific groups of study participants who possessed the characteristics of, or live in settings relevant to, the social phenomenon under study, in this case students in their late adolescence living in a university campus setting.^25,26^ Data collection was in the form of audio-recordings of face-to-face interviews with study participants on the research subject using a validated interview guide. Duration of engagement with the study participants through the interview sessions was sufficiently long. The recorded conversations, including nonverbal observations, were transcribed verbatim by the two study authors by single transcription approach. Transcribed data were stored in a secure personal laptop computer. Meticulous records of the interview conversations and observations, together with documentation of the process of analysis in detail, assured credibility of the data analysis.^25^ An independent review of both the audio recordings of the interview sessions and the verbatim transcripts of the interviews was performed by the dissertation advisor who supervised the study. Furthermore, the audio-recordings and verbatim transcripts were validated by the University of Liverpool, United Kingdom, for a Master’s degree in Public Health dissertation. The documentation of the entire research process and the outcomes were detailed and represent a publicly accessible representation of the interactions under investigation, which can allow other researchers to evaluate the data with respect to its confirmability, dependability and transferability.^25^

### Reflexivity

Throughout this study, the researcher maintained a detached attitude and open mind to the responses of the study participants. He brought neither personal theoretical assumptions to the research question nor did he exhibit any form of curiosity towards the views and opinions expressed by the study participants.^26,27^

### Data management and analysis

In this study, thematic content analysis was carried out on transcribed data.^28^ The first step in the data analysis in this study involved verbatim transcription of interview data. In the second step, codes that represent the basic elements of the raw data related to the study objectives that can be meaningfully assessed were manually developed from the data by the researcher. The third step involved searching for themes, that is, organising different codes into potential themes. In the fourth step, the generated themes were defined and labelled with respect to the study aims and objectives and the aspects of the data captured by each theme. The fifth step was the generation of the analysis report that provided a coherent and logical account of the data story as captured from the set of fully developed themes, with sufficient data extracts to show the prevalence of the generated themes (Table 2).

**TABLE 2:** Themes, codes and categories.

Study objectives	Themes	Codes and categories
Perceptions of healthy lifestyle	1.1. Balanced diet.	1.1. Good diet, fruit and vegetable consumption, adequate water intake, carbohydrates, proteins, fats and oils, minerals.
	1.2. Regular physical activity.	1.2. Physical exercise, physical fitness, obesity prevention, mental alertness, endurance, resilience.
	1.3. Disease prevention.	1.3. Absence of disease, personal and dental and environmental hygiene, medical check-up, therapeutic interventions, adequate rest.
Attitudes towards healthy lifestyle	2.1. Personal conviction.	2.1. Dietary choices, religious belief (fasts).
Practices towards healthy lifestyle	2.2. Personal disposition.	2.2. Junk food consumption.
	3.1. Meal skipping.	3.1. Breakfast skipping, lunch skipping, religious fasts.
	3.2. Junk food consumption.	3.2. Frequent, infrequent, high-fat diet, cakes, sugar drinks, energy drinks, meat pies, poor dental health, school environment, personal disposition.
	3.3. Fruit and vegetable consumption.	3.3. Frequent, infrequent, regular, irregular, personal disposition, home environment.
	3.4. Regular physical activities.	3.4. Aerobic and anaerobic exercises, domestic chores, staircase climbing, pedestrian commuting.
Influences on practices towards healthy lifestyle	4.1. Academic schedules.	4.1. Meal skipping, junk food consumption, school environment.
Influences on attitudes towards healthy lifestyle	4.2. Peers.	4.2. Junk food consumption, sporting activity, fruit and vegetable intake, fast food restaurant patronage.
	4.3. Socio-cultural background	4.3. Religious belief, religious fasts, family and home environment, high carbohydrate diet.
	4.4. Secondary school background	4.4. Public, private or mission school; peers; compulsory sporting activity; optional sporting activity; pedestrian commuting; school cadet membership.
	4.5. Financial resources	4.5. Dietary choices, junk food consumption, patronage of fast food restaurants, self-prepared meals.
	5.1. Parents.	5.1. Parents’ view of fast foods.
	5.2. Fast food quality concerns.	5.2. Patronage of fast food restaurants, environmental hygiene of fast food restaurants, personal hygiene of caterers.
Practices towards sedentary activity	6.1. Television viewing.	6.1. Frequent, infrequent, length of time for TV viewing.
Influences on practices towards sedentary activity	6.2. Computer and Internet activity.	6.2. Frequent, infrequent, length of time for computer and Internet activity, academic activity.
	7.1. Academic activity.	7.1. School work, access to TV andcomputer, information seeking.
	7.2. Home environment.	7.2. Social networking, electric power supply, leisure.

TV, television.

### Ethics statement

The study was approved by the LASU Teaching Hospital Health Research and Ethics Committee. In addition, the authorities of the two universities from which the main study participants were recruited, the LASU and Caleb University approved the recruitment of their respective students as participants. As this study was based on a dissertation by the lead author titled ‘Perceptions, Attitudes and Practices of adolescents in Lagos, Nigeria towards Healthy Lifestyle: A Qualitative Study’, for the award of Master of Public Health degree of the University of Liverpool, United Kingdom, the institution’s Research Ethics Committee also approved the study.

## Results

Each of the 18 study participants is identified by a participant number: T1–T2, P1–P16 and an alphanumeric label (Table 1). The thematic index^26^ (Table 3) summarises the key findings in this study. Twenty-one themes were constructed from the study analysis in line with the study objectives (Table 2).

**TABLE 3:** Thematic index.

**How study participants perceived healthy lifestyle** 1.1. Balanced diet (e.g. fruit and vegetable consumption, adequate water intake, carbohydrates, proteins, fats and oils, minerals). 1.2. Regular physical activity (e.g. physical exercise, physical fitness, obesity prevention, mental alertness, endurance, resilience against physical stress). 1.3. Disease prevention (e.g. personal and dental hygiene, environmental hygiene, periodic medical check-up, therapeutic interventions, adequate rest). **Study participants’ attitudes towards healthy lifestyle** 2.1. Personal conviction (e.g. dietary choices, religious beliefs and fasting). 2.2. Personal disposition (e.g. consumption of cakes, sweetened drinks, energy drinks). **Influences on study participants’ attitudes towards healthy lifestyle** 3.1. Parents (e.g. parents’ view of fast foods). 3.2. Fast food quality concerns (e.g. environmental hygiene of fast food restaurants, personal hygiene of caterers). **Study participants’ practices towards healthy lifestyle** 4.1. Meal skipping (e.g. breakfast skipping, lunch skipping, religious fasts). 4.2. Junk food consumption (e.g. frequent, infrequent, regular, irregular, high-fat diet, cakes, sugar drinks, energy drinks, meat pies, poor dental health). 4.3. Fruit and vegetable consumption (e.g. frequent, infrequent, regular, irregular, home environment). 4.4. Regular physical activity (e.g. aerobic and anaerobic exercises, domestic chores, staircase climbing, active [pedestrian] commuting). **Influences on study participants’ practices towards healthy lifestyle** 5.1. School environment (e.g. active pedestrian commuting, academic schedule). 5.2. Peers (e.g. fruit and vegetable consumption, junk food consumption, fast food restaurant patronage). 5.3. Family and home environment (balanced diet, regular fruit and vegetable consumption). 5.4. Secondary school background (e.g. public, private or mission school; compulsory sporting activity; optional sporting activity; active pedestrian commuting). 5.5. Social-cultural background (e.g. religious fasts, high carbohydrate diet, fruit and vegetable consumption). 5.6. Financial resources (e.g. junk food consumption, fast food restaurant patronage, self-prepared meals). **Study participants’ practices of sedentary activity** 6.1. Television viewing (e.g. frequent, infrequent, length of time for TV viewing). 6.2. Computer and Internet activity (e.g. frequent, infrequent, length of time for computer and Internet activity, academic activity). **Influences on study participants practices towards sedentary activity** 7.1. Academic activity (e.g. school work, information seeking, access to TV and computer). 7.2. Home environment (e.g. information seeking, social networking, leisure).

TV, television.

These themes and their accompanying study objectives and the key responses of the study participants are described next.

### Study participants’ perceptions of a healthy lifestyle

Most study participants expressed their understanding of healthy lifestyles in terms of balanced diet, physical activity and physical fitness. Some of the study participants also perceived healthy lifestyles from the perspective of disease prevention or absence of disease, including medical check-ups and prevention of diseases through personal and environmental hygiene:

‘Healthy lifestyle, eh … mm, living like you are fit, strong health-wise; you take the right diet or food, do the right exercise, you keep fit.’ (P13, female, architecture student)

The study participants’ perceptions of healthy lifestyle are further captured by the following themes.

#### Balanced diet

Most participants viewed healthy lifestyles in terms of regular meals and eating a balanced diet:

‘I think it means, like, what’s happening in a day – you wake up in the morning, take healthy breakfast, as in a balanced diet, and eat lunch when lunch time is due, eat dinner when dinner time is due.’ (P15, female, architecture student)

#### Regular physical activity

All the participants perceived healthy lifestyle in terms of regular physical activity. However, their perceptions revealed different perspectives such as physical fitness, obesity prevention, mental alertness, enhancing self-confidence and building resilience against physical stress:

‘Physical activity, that’s exercise right? Yeah, exercises are quite good in order to live a healthy lifestyle, because exercise can help one to burn off fat, the unnecessary fat in the body, so exercise also can help keep the brain sharp and everything. Exercises are quite … they are good for the body in order to live a healthy life.’ (T2, male, preclinical medical student)

‘Physical activity, they help healthy lifestyle a lot as your body tends to be basically fit; there’s this confidence that it gives you that you can actually do something that other people can’t do; how will I put it? … When for even, even in your working place or even in class at times, you tend to – as in when there are classes, they are strenuous classes, you tend to endure it more as compared with other people.’ (P16, male, preclinical medical student)

### Disease prevention

Some study participants also described their understanding of healthy lifestyles in terms of disease prevention, such as therapeutic interventions, and in terms of personal and environmental hygiene. Some participants expressed the view that personal and environmental hygiene were important for a healthy lifestyle, suggesting an understanding of the concept of a healthy lifestyle as prevention of communicable diseases:

‘A healthy lifestyle to me means living the right way in a clean environment, living in an area that is without, eh … mm, against disease; you can live peacefully without having diseases, that’s healthy lifestyle; living a clean lifestyle.’ (P3, male, law student)

‘When you have headache, take the appropriate drug, take enough rest, enough exercise, go to hospital for check-up and the like.’ (P8, female, English language student)

Another participant stressed the importance of regular medical check-ups as a means of maintaining a healthy lifestyle:

‘I feel one should always have regular check-up to know if he or she is still healthy enough.’ (P14, female, mass communication student)

### Study participants’ attitudes towards a healthy lifestyle

Analysis of the study participants’ attitudes towards a healthy lifestyle illuminated two key themes, namely personal conviction and personal disposition in relation to junk food consumption:

‘They say don’t eat this or don’t eat that and it just; I just think it’s like they say what you know won’t kill you; so if I know eating this won’t kill me I can just keep eating it and feel healthy.’ (P10, female, architecture student)

‘I’m a snack kind of person; because sometimes I just don’t really like food, something just … I’ll just prefer going for a bottle of any of these drinks and things like egg roll or meat pie or something like that.’ (P3, male, law student)

### Influences on attitudes towards a healthy lifestyle (dietary habits)

Personal concerns about fast food product quality and parents’ views of fast food were the important themes here.

#### Fast food quality concerns

Some study participants had concerns about the quality of fast food products, as illustrated by the following data extract quote:

‘Well, I like fast food joints but I try not to frequent the food because I don’t know how it was prepared. It’s only when I’m very, very hungry and I’m feeling too lazy to cook … that’s when I go there.’ (P1, female, preclinical medical student)

#### Parents’ view

This had an important influence in the context of fast food consumption for some of the study participants. One study participant narrated her experience with parental influence on her attitude towards fast food consumption:

‘Fast food joints, eh … mm, I like it, but I don’t have the opportunity to take it because of my parents … because they say it’s not good for the body, it’s not right and all … yeah.’ (P14, female, mass communication student)

### Study participants’ practices towards healthy lifestyles

Study participants’ practices towards healthy lifestyles were illuminated by four key themes: meal skipping, junk food consumption, fruits and vegetables consumption and practice of regular physical activity.

#### Meal skipping

Most participants described a dietary practice of meal skipping, especially in the school environment, with breakfast and lunch as the typically skipped meals:

‘Sometimes, my breakfast [*implying first meal in the day*] would be in the afternoon because I’m very busy in class, and sometimes I may not eat till in the evening; that’s because of the time we have to be in class, so school is a major factor.’ (T1, female, physiology student)

#### Junk food consumption

Study participants engaged in the practice of junk food consumption, with most participants doing so frequently. Junk food was typically in form of locally made snacks such as meat pies, egg rolls, cakes and sweetened sugar and energy drinks. Interestingly, some study participants expressed the notion that sweetened sugar drinks provided an important energy source for sustaining their highly active student lifestyle:

‘Someone like me loves eating those things [*referring to junk snacks*] a lot, because as a student you burn a lot of calories, so you need all these sugary stuff. Like when you are trekking from a place of lecture to another place, like you are burning a lot of calories and you need more blood sugar in your body, so you have to take lots of these things.’ (P9, male, history and international relations student)

One of the study participants pointed out that sugary drinks and confectioneries are bad for dental health:

‘I really don’t take those things such as yoghurt, chocolate or sugary drinks because they are not good for the teeth, they are not good for health; I only take *gala* [*beef sausage*] when I’m broke.’ (T1, female, physiology student)

### Fruit and vegetable consumption

All the study participants perceived regular consumption of fruits and vegetables as part of healthy lifestyle; while some of them consumed fruits and vegetables on a frequent and regular basis, mostly in the home environment, most did not:

‘I take them a lot. I take especially fruits – apples, oranges, pineapple – on a daily basis because I think these fruits really have the right vitamins. I need Vitamin C, so I try to take it as much as possible. I also take vegetables on a regular basis, because they are also rich in minerals and stuff.’ (P7, male, computer science student)

‘Fruits, as I heard, are really good for the body … when you take fruits … vegetables … it gives you more blood, but there are some fruits that give you stronger teeth such as carrots … eh … mm … sorry, that’s vegetable … then apple … then you have … hmm … in a nutshell. I just think … I think it has more importance … or more advantage than taking junks. Taking junk food will obviously cause more harm than good.’ (P11, female, mass communication student)

#### Physical activity

The practice of regular physical activity such as physical exercise, staircase climbing, daily domestic chores and active pedestrian commuting was common among study participants. Active pedestrian commuting was in particular a common practice of all participants, especially within the school environment:

‘I’m moving from one faculty to another faculty, walking down. I come from home, so I walk to my bus stop and take a bus to the school gate and then walk from the school gate to my faculty.’ (P4, male, law student)

### Influences on study participants’ practices towards healthy lifestyles (dietary habits)

The main influence on participants’ practices towards a healthy lifestyle in the context of dietary habits were elucidated by the following themes: academic schedules, peers, sociocultural background, family, secondary school background and financial resources.

#### Academic schedule

This influence was mainly in the school environment and applied to dietary practices of participants. The participants’ busy academic schedules often made them skip their breakfast and lunch:

‘Yeah, I skip lunch, not all the time, because when I go to class, because of classes and all that, I used to skip lunch and sometimes breakfast, but I make sure I take dinner.’ (P5, female, microbiology student)

#### Peers

The influence of peers on the practice of healthy lifestyle was described in relation to dietary habits of some study participants:

‘Personally I learnt from M [*first name initial of his colleague*] that I should take fruits maybe once in 3 days. I’ve learned that it is better to have fruits twice in a day because even aside from helping your bowel, it helps at times to regulate the blood pressure, the heartbeat, so yes, it is a major influence on me.’ (P6, male, computer science student)

#### Sociocultural background

Cultural background and religious background were perspectives under this theme. Cultural background was perceived as a determinant of dietary habits in many of the study participants, while religious background was a determinant of dietary practices in some of the study participants, who narrated their observation of religious fasts in relation to meal skipping:

‘I think what I heard is that, eh … mm, or because of research, it said something like when you fast often, you won’t – like your health would be something else, like you will have a good health, so sometimes I do fast during the day and used to break my fast around six [*18:00*] and just to improve myself.’ (P9, male, public university student)

‘The religious background, I think that is actually one of the factors that has influenced me that much, because most times we do a lot of fasting, although most of them are voluntary, but you just want to join so I do it too, when you have 3 days in a week.’ (P13, female, private university student)

#### Family and home environment

This had an important influence on the dietary habits of most study participants in the context of the home environment:

‘When I’m at home it is quite easy eating balanced diet, taking my three square meals, but compared with school, as I said before, it’s quite hard keeping up with three square meals, a balanced diet.’ (P4, male, law student)

‘But most of the time, my mum gives me – like I actually adopted it from my mum. She loves taking fruits and actually when she is taking it, I join her; that’s how I developed a habit of taking fruits.’ (P8, female, English language student)

#### Secondary school background

Type of secondary school (military school, mission school, private school, boarding school) were perspectives under this theme:

‘They [*her secondary school*] taught me always in class how to be healthy, about balanced diet and what to eat and what not to eat….’ (P11, female, mass communication student)

‘Hmmm … I think my secondary school went a long way in helping me in my healthy lifestyle, of course. One, we were not allowed to bring something [*food, beverages*] to school, so that helped me to get away from eating in eateries. My body got used to eating smaller meals, and in fact I was very slim [*expresses bemusement*] in secondary school because there was no extra sugar or extra anything. They [*the school*] gave us food, of course, they gave us provisions sometimes, but just a very eh … mm … minimal … very minimal rate. Eh … it was all boarders [*boarding school*].’ (P2, female, preclinical medical student)

#### Financial resources

This theme emerged as an influence on dietary habits of study participants, whereby a lack of or inadequate financial resources influenced dietary choices in some of the study participants. Inadequacy of financial resources was related to a tendency to go for junk food rather than regular meals: ‘I might be looking at buying food outside or I just buy snacks such as drinks for money issues’ (P3, male, law student).

### Influences on study participants’ practices towards healthy lifestyles (physical activity)

The theme that emerged under this context was secondary school background.

#### Secondary school background

The perspectives under this theme include type of secondary school (i.e. public, private, mission, military), regular sporting activities, active pedestrian commuting. All the participants expressed the view that their secondary school background influenced their practices towards physical activity. The perspectives are reflected in the following extract:

‘Well … what I remember is … it’s much fun; hmm … let’s say Monday, nothing much. Tues[*day*] … Wednesday, basically, we take part in sports, as in we close on time; I think around two o’clock. Yeah, we close around two [*14:00*] and do sports till around three … four [*15:00–16:00*]. We at times … running [*athletics sport*]. I was never even in the football team, so I never even liked football then.’ (P16, male, preclinical medical student)

### Study participants’ practices towards sedentary activity (physical inactivity)

Television viewing and computer and Internet activity were the key themes in this context, with the latter as the predominant sedentary activity study participants engaged in. Television viewing was a less frequently practiced sedentary activity.

#### Study participants’ practices towards television viewing

Most participants described an infrequent TV viewing practice, which they considered to have been superseded by the computer and Internet. This is illustrated by one participant’s description of her practice:

‘I hardly watch TV because right now, I don’t think anyone likes to watch TV, because I don’t see why I should watch TV when I can get it on my phone, when I can get it on my laptop. A lot of people are sitting down on the chair and watching something very far from them when I can be into it with my laptop or check any information with my phone.’ (P11, female, mass communication student)

#### Study participants’ practices towards computer and Internet activity

This was a very common sedentary activity practiced by all study participants, who frequently spent time on the computer and Internet, which they engaged in both for social networking and academic activity, as illustrated by the following narrative:

‘I don’t have time again because of Internet, Internet – like you get to know what’s happening around the world, views or comments on upcoming events and all, like everywhere around you – Internet. Internet takes you round anything you need to know, you can just go to the Internet, Google it up and you see it; so I spend more time on the Internet.’ (P14, female, mass communication student)

#### Influences on study participants’ practices towards sedentary activity (physical inactivity)

This theme gave an insight into the factors that influenced sedentary behaviour among study participants. Academic activity (in respect of schoolwork and seeking knowledge and/or information) and home environment (in respect of leisure and social media interactions) represented key influences.

#### Academic activity

While academic activity was associated with school environment, the relative physical inactivity in the home environment favoured leisure-related sedentary activity:

‘I use the Internet mainly for school work and maybe social media, eh … mm … Twitter, majorly, or WhatsApp. Apart from these, maybe to keep up to date with what’s happening around the world, just to chat with friends, find out what’s going on around the world and school.’ (P6, male, computer science student)

#### Home environment

‘Maybe that’s because I’m at home, but when I’m in school I get to go up and down [*physical activity*], read book, do Internet surfing; I think at home, I’m usually very inactive [*physically speaking*], not really busy at home.’ (P14, female, mass communication student)

‘Well, I do that [*sedentary activity*] more at home. I don’t really like […] I do that more at home, unlike in school. At home, I don’t really have to go out, unlike in school, if I need to see someone I have to go out [*doing some form of physical activity*]. If I need to eat something, I have to go out and buy it [*doing some form of physical activity*]. So, I stay [*implying less physical activity*] more at home than in school.’ (P1, female, preclinical medical student)

## Discussion

In this study, study participants perceived a healthy lifestyle as a balanced diet, regular physical activity and disease prevention. Their attitudes and practices towards healthy lifestyles were described in the context of personal conviction and disposition, regular physical activity and consumption of fruits and vegetables. Meal skipping and consumption of junk food were observed as unhealthy dietary practices. Family, home environment, the school campus physical environment, peers, secondary school background and concerns about fast food quality were highlighted as positive influences on healthy lifestyle, while academic schedules, personal disposition, school campus fast food eateries and sociocultural background were negative influences. Computer and Internet activity was the main sedentary activity.

While a healthy lifestyle has been universally conceptualised in the context of patterns of dietary intake, physical activity and inactivity, tobacco smoking and alcohol consumption as central to evidence-based guidelines for diverse populations,^2^ this study focused on patterns of dietary intake, physical activity and sedentary behaviour (physical inactivity). Patterns of tobacco smoking and alcohol consumption are more variable and less common indices for evaluating healthy lifestyle in the adolescent population in Nigeria.^19,20,21^

Study participants’ perceptions of healthy lifestyles in the context of balanced diet and regular physical activity were consistent with the evidence-based guidelines for the concept of healthy lifestyles.^2^ However, their perception of healthy lifestyles in terms of disease prevention did not align with the guidelines. Nevertheless, their recognition of personal and environmental hygiene as the basis for disease prevention reflected an understanding of an important public health problem in the study environment, the challenge of communicable diseases.

Again, their perception of a healthy lifestyle in relation to a balanced diet was described in the context of fruit and vegetable consumption, adequate water intake and having the appropriate complements of carbohydrates, proteins, fats and oils and minerals. This was in agreement with the perception of female adolescent students from a rural setting in South Africa who viewed healthy dietary practices as consumption of locally grown traditional foods, especially fruits and vegetables.^12^

The study participants also described their understanding of a healthy lifestyle in the context of physical activity such as regular physical exercise and physical fitness. Furthermore, they associated physical activity with obesity prevention, mental alertness, endurance and resilience against physical stress.

The study participants’ attitudes towards healthy lifestyles were elucidated in the context of personal convictions towards dietary choices and religious beliefs such as observation of religious fasts by some study participants. Some study participants’ attitudes towards a healthy lifestyle were also reflected in their personal disposition towards consumption of cakes, sweetened drinks and energy drinks. Some of them believed that sweetened sugar and energy drinks and confectioneries provided an energy source for sustaining their highly physically active student lifestyles in the university campus. This misconception encouraged unhealthy dietary practices. On the other hand, one study participant described a healthy disposition to regular consumption of fruits and vegetables because he believed fruits and vegetable are rich in minerals and other healthy elements.

With respect to practices towards healthy lifestyles in relation to dietary habits, although study participants associated regular consumption of fruits and vegetables with healthy lifestyles, the practice of regular consumption of fruits and vegetables by most of the study participants was largely infrequent and irregular, especially in the school environment compared with the home environment. This study also found unhealthy dietary practices that were inconsistent with a healthy lifestyle among the study participants in the form of meal skipping, such as breakfast skipping or lunch skipping, usually because of busy academic schedules and religious fasts. Meal skipping could lead to binge eating at the next meal, with potential consequences of overweight and obesity. Studies among American adolescents showed that breakfast consumption during school years was associated with a significant reduction in likelihood of developing obesity in later years.^4^

It was also observed that frequent consumption of junk food such as cakes, sugar drinks, energy drinks and meat pies was common among study participants. Excessive intake of free sugars has been associated with weight gain and obesity, with attendant increased risk of developing cardiovascular diseases such as hypertension, diabetes and metabolic syndrome.^6,7^ The built environment in the context of ubiquitous junk food eateries in the university campuses in the study environment promoted easy access to unhealthy dietary options.^29^ The frequency of junk food consumption by most of the study participants ranged from three to five times a week to several times in a day. Excessive fast-food consumption has been defined as a frequency of two times in a week.^29^ Canadian adolescents who live in neighbourhoods with a moderate or high concentration of fast-food restaurants were observed to be more likely to be excessive fast food consumers, compared with those living in neighbourhoods that lacked fast food restaurants.^29^

Some study participants recognised that sugary confectioneries and sweetened drinks were deleterious to oral health. A study in Hong Kong showed a longitudinal association between central obesity and dental caries in 15–18-year-old adolescents.^9^ Central obesity can result from frequent consumption of high sugar-containing confectioneries and drinks. On the other hand, studies have shown a correlation between healthy diet and good oral health.^30,31,32^

Frequent consumption of fast foods, high sugar-containing confectioneries and sweetened energy drinks, as observed in study participants, can lead to frequent lowering of the oral pH, making it conducive for bacterial attack,^30,31^ with resultant development of dental caries and periodontal disease. According to the World Health Organization, dental caries are a major global public health problem and the most widespread noncommunicable disease (NCD).^32^ It was found as the most prevalent condition in the 2015 Global Burden of Disease Study, with an estimated 2.3 billion people suffering from dental caries involving permanent teeth, with adolescents and children most at risk.^32^ Moreover, severe dental caries are a frequent cause of school absenteeism.^32^ The World Health Organization recommends improving the food environment in public institutions, especially schools, through regulation of sales of foods and beverages high in free sugar.^32^

Study participants also engaged in the practice of regular physical activity in various forms such as aerobic or anaerobic exercises, domestic chores, staircase climbing and active (pedestrian) commuting. This was consistent with a healthy lifestyle. Similarly, adolescent girls in rural South Africa actively took part in various physical activities including active commuting over long distances to school, domestic chores, traditional dances and outdoor activities.^12^

This study highlighted several influences on study participants’ practices towards the practice of a healthy lifestyle. The physical environment in the university campuses in this study, with long walking distances separating halls of residence from lecture halls and eateries, encouraged frequent active commuting by study participants on a daily basis, thereby promoting a healthy lifestyle. This was in alignment with the findings from a quantitative study of secondary school adolescents in Maiduguri, north-east Nigeria, which found that proximity of schools, libraries and shopping centres to adolescents’ homes and availability of necessary infrastructures for physical activity and walking were positive influences on active commuting to school by male study participants.^10^

On the other hand, busy academic schedules in the school environment facilitated unhealthy dietary practices such as breakfast and lunch skipping. A qualitative study of Iranian adolescent students similarly showed prioritisation of academic activity as a barrier to healthy lifestyle.^33^

Other influences on healthy lifestyle practices were home environment and peers who positively influenced a healthy dietary practice of regular intake of fruits and vegetables. The perceived influence of parental control on the dietary behaviour of young university students was similarly observed in a Belgian study.^16^ Furthermore, a study in Botswana found that adolescents’ perceptions of diet, physical activity and obesity were influenced by school activity, home or school environment, shopping mall and family and peers.^13^

Religious and social norms have been found to be barriers to female adolescents’ participation in outdoor physical activity.^34^ In this study, religious and cultural influences were limited to dietary practices such as meal skipping (religious fasts) and traditional high-carbohydrate diets such as pounded yam, rather than on physical activity.

Again, in this study the secondary school background was elucidated as an influence on study participants’ healthy lifestyle practices in the context of physical activity and dietary habits. This was reflected in regular compulsory sporting activities and discouragement of unhealthy dietary practices. This observation differed from the findings of quantitative studies among secondary school adolescents in Nigeria, which found a low level of physical activity and unhealthy dietary practices among most study participants, especially those from private schools.^9,10^

Lack of financial resources was another negative influence on the practice of healthy lifestyles illuminated in this study, as it influenced the dietary choices of some of the study participants towards unhealthy junk foods. Similarly, scarcity of financial resources was observed as a barrier to healthy lifestyle in a study of adolescent students in Iran.^33^

Study participants frequently engaged in computer and Internet sedentary activity for academic purposes such as school work and information (knowledge) seeking, as well as for social networking and leisure in the home environment, with varied frequency and length of time spent on the activity. A study in China^35^ found that the use of the Internet as a means of gaining knowledge or obtaining information was predictive of healthy lifestyle in adolescents.

## Strengths and limitations of the study

The meticulous records of the interview conversations and observations through the verbatim transcription process, together with the documentation of the process of analysis in detail, assured credibility of the data analysis.^25,26^ Moreover, an independent review of both the audio-recordings of the interview sessions and the verbatim transcripts of the interviews was performed by a research advisor who helped to supervise the study.

A similar study using quantitative methods could serve to triangulate the findings and enhance the credibility of this study.

## Recommendations

The authors recommend that regulatory policies should be enacted by relevant authorities to ensure that eateries and other food vendors operating in university institutions provide only healthful dietary options. This is in alignment with the World Health Organization’s recommendation of improving the food environment in public institutions through regulating sales of foods and beverages high in free sugar.^32^ Furthermore, breakfast break periods should be established in university institutions to curb the widespread unhealthy practice of breakfast skipping among adolescent university students occasioned by busy academic schedules.

## Conclusion

Study participants’ perceptions of healthy lifestyles in terms of balanced diet and physical activity were consistent with the evidence-based guidelines for concept of healthy lifestyles. Their practices towards healthy lifestyles reflected a high level of physical activity in the university campus environment, facilitated by the physical environment. However, study participants’ dietary practices, while largely consistent with a healthy lifestyle in the home environment, were characterised by unhealthy practices in the university environment facilitated by busy academic schedules and the ubiquitous presence of junk food eateries in the university campuses. Future research is suggested to explore the benefit of study participants’ engagement in computer and Internet sedentary activity for academic purposes and knowledge seeking towards healthy lifestyles.
